# Psycho-emotional acceptance in couple and single women who choose to undergo IVF treatment with donor eggs

**DOI:** 10.5935/1518-0557.20220036

**Published:** 2023

**Authors:** Helena L Montagnini, Carolina T. Kimati, Aline R. Lorenzon, Tatiana CS Bonetti, Paulo C Serafini, Eduardo LA Motta, Thais S. Domingues

**Affiliations:** 1 Huntington Medicina Reprodutiva - Eugin Group. Av República do Líbano, 529 Ibirapuera. São Paulo, SP 04501-000, Brasil; 2 Disciplina de Ginecologia Endocrinológica, Departamento de Ginecologia, Escola Paulista de Medicina da Universidade Federal de São Paulo (UNIFESP-EPM). Rua Napoleão de Barros, 608 Vila Clementino, São Paulo, SP, 04024-002, Brasil; 3 Disciplina de Ginecologia, Departamento de Obstetrícia e Ginecologia, Faculdade de Medicina, Universidade de São Paulo (USP). Av. Dr. Arnaldo, 455 Cerqueira César, São Paulo, SP 01246-903, Brasil

**Keywords:** Oocyte Donation, Emotional State, *In Vitro* Fertilization

## Abstract

New family configurations are emerging concurrently with improved assisted reproduction techniques, including the use of donated gametes. Most indications for treatment when using donated eggs are caused by an age-related decrease in reproductive capacity. We evaluated the emotional state regarding accepting egg donation in participants who chose this option for in vitro fertilization cycles. This is a retrospective, Brazilian cohort study, based on data collected from sixty psychological counseling sessions with participants that opted to be enrolled in an egg donation program. A single professional conducted semi-structured psychological counselling sessions. The data were analyzed using a thematic analysis as the qualitative methodology. Two years after the psychological counseling sessions, participants were contacted to obtain information about their outcomes. Of 60 sessions, 19 (32%) were classified as involving participants with positive emotional state (group 1), 14 (23%) with unfavorable emotional state (group 2), and 27 (45%) without evident classification (group 3). Three couples did not undergo treatment until two years after the psychological counselling session and the other couples underwent treatment in a period ranging from 1-8 months after the session. This is the first study in the Brazilian population regarding the acceptance of egg donation. The process of acceptance of infertility and the impossibility to have a biological child is fundamental to gradually accepting a new way of becoming a parent. Psychological counseling can contribute to reflecting on the use of donated eggs, exploring its emotional implications and identifying the need for psychotherapeutic work to address conflict and suffering.

## INTRODUCTION

For couples who desire to have a child and are unable to conceive naturally, infertility is a painful and often emotionally devastating condition (Schaffer & Diamond, 1994; Syme, 1997; Peluffo, 2008; Mahstedt *et al*., 2010, Blake *et al*., 2014; Hammond, 2018).

Despite social changes that have occurred in recent decades, maternity continues to be desired and valued by many women as an important aspect of female identity. Some women refer to a sense of incompleteness because they do not have children and thus feel stigmatized by a society that expects them to be mothers (Montagnini & Lopes, 2015; Hammond, 2018). However, maternity is increasingly being postponed, as it is not the only source of fulfillment for women. Professional and academic development, as well as financial stability, are valued and desired, and they require dedication, which sometimes is difficult to reconcile with motherhood (Hewlett, 2002, Montagnini & Lopes, 2015).

The increase in life expectancy and the consolidation of conjugal affective relationships later in life also contribute to postponing parenthood. However, when couples decide to have a baby, they are sometimes faced with difficulties and limitations that conflict with the expectation that pregnancy will occur at the desired time. Faced with the impossibility of becoming pregnant, couples have alternatives, including seeking medical treatment to overcome this difficulty. If couples choose this alternative, they undergo a process that will affect various aspects of their lives and can have intense emotional repercussions (Peluffo, 2008; Benyamini *et al*., 2009; Blake *et al*.,2014).

Using donated eggs is indicated when the woman’s reproductive capacity is lost or decreased. This condition is mostly associated with woman’s age, as oocyte quality and quantity start to decrease after 30 years old and most significantly after 35 years old (Igarashi *et al*., 2015). Through in vitro fertilization treatment using donated eggs, donor eggs (from a women under 35 years old) are fertilized in the laboratory with the husband’s semen, and the developed embryo is transferred to the woman’s uterus (recipient). Single women, after medical indication to use donated eggs, also need to undergo semen donation.

Laws and regulations of egg donation programs vary in different countries, which impacts the results and conclusions of studies carried out on this topic (Bracewell-Milnes *et al*., 2016). In Brazil, until May, 27^th^ 2021,according to the Federal Council of Medicine resolution, gamete donation is anonymous, voluntary, and can be neither profitable nor commercial. However, from that date onwards, it is possible to donate gametes between relatives of up to 4th degree of one of the recipients, as long as there is no consanguinity In exceptional situations, information about donors, for medical reasons, can be provided exclusively to physicians, safeguarding the donor’s civil identify in anonymous donation (Resolution CFM 2.168/2017). In France, Denmark and Spain, gamete donation is also anonymous (Hammarberg *et al*., 2008). In other countries, such as the United States, the identity of the gamete donor can be disclosed and the practice can be remunerated (Sabatello, 2015). In other countries, such as Sweden, Norway, Austria, Switzerland, the Netherlands, New Zealand and UK, donor identity can be disclosured upon request from the offspring after reach adulthood (Golombok *et al*., 2011).

Matching the egg donor and the recipient (including phenotypic characteristics, when possible) is the responsibility of the doctor in Brazil. Egg donor are always young women (until 35 years old), who decide to either donate in an altruistic behavior or when undergoing an IVF treatment for male infertility. In this case, the recipient covers the donor costs of ovarian stimulation (called shared egg donation). The waiting time for egg recipients varies between 3 to 6 months in our service.

Egg donation has occurred in Brazil for approximately 25 years, but this possibility has been more openly spoken about in press and social media only in the last five years, contributing to an increased knowledge in the general public (Montagnini *et al*., 2012). However, this has happened in a heterogeneous way, considering marked regional and economic differences in Brazil. Infertility treatments, including egg donation, are mostly done in private health services, and the costs are expensive. There are few public services for assisted reproduction treatments in Brazil, and the government rarely pays for the medications. Despite the popularization of egg donation, the majority of patients who use donor gametes keep this information confidential, not favoring its visibility and social acceptance (Montagnini *et al*., 2012). Hammond (2018) emphasizes that the greater visibility given to egg donation lately contributes to personal and social acceptance of these families, favoring and encouraging more people to consider this alternative.

Several research studies reported that confidentiality regarding using eggs is in part motivated by the fear of social stigmatization of the woman and future child, as they are different from the standard family (Nachtigall *et al*., 1997; Shehab *et al*., 2008, Laruelle *et al*., 2011; Indekeu *et al*., 2013; Applegarth, 2016; Hershberg *et al*., 2019).

Indication for treatment using donated eggs is often received with surprise, even by women who are in their 40s (Sydsjö *et al*., 2014). The realization of a reproductive limitation contrasts with personal experiences of vitality, health, joy, achievement, success and professional productivity (Luk *et al*., 2010). While the women may feel young and capable, they are nevertheless faced with the biological consequences of ageing (Luk *et al*., 2010).

These limitations can have an emotional impact on women’s sense of self, with repercussions on self-esteem, which can cause feelings of incapacity, shame, guilt and inferiority due to the impossibility of having a child using their own gametes (Peluffo, 2008). These feelings of worthlessness are devastating to many women and a narcissistic wound that must be healed (Peluffo, 2008; Mahlstedt & Greenfeld, 1989; Mahlstedt *et al*., 2010; Sydsjö *et al*., 2014; Greenfeld, 2015).

Although it represents a limitation and a loss, egg donation points to a new alternative for having a child, as it may maintain the genetic connection with the father and the bond between mother and baby through gestation (Golombok *et al*., 2013).

In the cases that men infertility is not also present, they may preserve their biological link, however, accepting eggs from another woman does not always occur without conflict and suffering, as it may not be what they initially desired (Hargreaves, 2006).

After a long history of loss and unsuccessful treatments, many couples now consider egg donation a source of hope in realizing their desire to become pregnant and have a child (Peluffo, 2008). However, they may experience mixed feelings of pain and hope and have contradictory thoughts while undergoing the process of deciding whether or not to receive eggs from a donor (Peluffo, 2008).

During this process, couples do not always have the opportunity to reflect on the procedure’s implications because the attention is focused on the desire to solve the infertility issue and not on the accompanying emotional distress. Only after the child is born do questions about the treatment process and its repercussions in their lives and larger family relationships arise (Schaffer & Diamond, 1994).

The multiple types of losses coped by this patients, from miscarriages to the loss of fertility, have potential implications in future family relationships (Mahlstedt, 1985). These losses refer to both objective (miscarriages) and especially, subjective losses. Subjective losses are those that have no visibility, such as loss of the possibility of having a desired biological child, loss of the possibility of transmission of family genetics to the child, loss of the fertile body, self-esteem and spontaneity in sexual life (Mahlstedt, 1985). Considering that, several authors emphasize the importance of the psychological preparation for couples who require treatments with donated gametes (Berger *et al*., 1986; Mahlstedt & Greenfeld, 1989; Nachtigall *et al*., 1997, Syme,1997; Peluffo, 2008; Mahlstedt *et al*., 2010; Laruelle *et al*., 2011; Blake *et al*., 2014; Benward *et al*., 2015; Greenfeld, 2015; Hershberger *et al*, 2021).

This study’s objective is to evaluate the emotional state regarding accepting egg donation by couples and single women who chose to use donated eggs in a specialized assisted reproduction clinic in São Paulo, Brazil.

## Materials and Methods

### Participants

This is a retrospective, Brazilian cohort study based on data collected from sixty psychological counseling sessions with participants (43 couples and 17 women, of whom 4 were single) that opted to be enrolled in an egg donation program between September and December 2014 in a private clinic in São Paulo, Brazil, after receiving medical indication. This represents 54.54% of patients who joined the egg donation program in the clinic during this period. In Brazil, psychological counseling sessions for patients that will use donated gametes for conceiving is not mandatory, however we offer a session free of extra charge to all patients that is enrolled to the program.

The service’s protocol included an optional psychological counseling session to take into account the questions and emotions for couples facing these choices, the decision-making processes in using donated eggs and the exploration of the emotional implications of gamete donation. The couple’s participation is recommended, considering both are involved in the proposed treatment.

This study was approved by the Ethical Committee of Hospital da Força Aérea de São Paulo - HFASP (CAAE 12900319.7.0000.8928).

### Semi-structured sessions

Semi-structured sessions were conducted with participants with the objective of discussing topics of interest regarding in vitro fertilization using donated eggs, the decision-making process and the exploration of the emotional implications of gamete donation.

Psychological counselling session were conducted using questions designed to elicit participants’ reactions to experiences related to the addressed topics and configured according to participants’ specific demands and needs. This characteristic implies a heterogeneity of the sessions and data obtained.

When conflicts concerning the use of donated eggs are identified, indicating that the psychological acceptance of the treatment is still not entirely assured, follow up is recommended to give an opportunity to start the treatment with greater safety and tranquility. In these cases, the therapist’s choice is made at the patient’s discretion, being also possible to continue with the clinic’s psychologist if the patient desires.

This study was developed based on data concerning the decision-making process in using donated eggs, according to patient’s statements during the psychological counselling sessions, considering its relevance to the emotional state, to initiate the assisted reproduction treatment.

### Procedures

After medical indication to use donated eggs, participants scheduled an appointment with a nurse to obtain detailed information about the egg donation program.

The optional psychological counseling session is offered to these patients. The sessions lasted approximately 60 minutes and were performed by a single psychologist who is specialized in infertility.

The same psychologist analyzed the data set using a thematic analysis as the qualitative methodology (Minayo, 2007). Thematic analysis is an analytic method to identify and analyze themes and patterns of meaning in a data set (Clarke & Braun, 2014).

Brief notes (topics covered, words and expressions used, dates, history of infertility and treatments performed) were taken during the psychological counseling sessions in order to facilitate later data registration. Immediately after session completion, a detailed record of the content covered was manually transcribed, as well as the way it was delivered and the feelings that were evidenced. When the sessions were with couples, the transcription highlighted both points of view. A summary of the session was also typed and register at the patient’s electronic medical record. Recorded material was read, analyzed and classified according to themes, which consist of statements about certain subjects. The selected themes were as follows: history of infertility (diagnosis, treatments performed and referred feelings); losses (miscarriages or of born children); decision-making process (feelings and thoughts regarding treatment with donated eggs, coping strategies, feelings and thoughts when entering the program, and time elapsed between these two moments); egg donation (references and questions related to egg donation, especially regarding the donors and the mother-child relationship); and marital relationship (references made to the marital relationship). From this data categorization, participants were classified retrospectively in three groups according to emotional states related to proceeding with treatment. For the classification, the couples sessions were considered as a unit, but differences and divergences were considered in the classification. Couples considered to be in favorable emotional states were those in which both members were assessed in this way. The characteristics of each group are described below.

Group 1: Participants exhibiting favorable emotional state. In this group, participants demonstrated acceptance of the impossibility of using their own eggs, or the partner’s eggs in the case of men, and receiving donated eggs. There was a period in which feelings of frustration resulting from the impossibility of having a biological child, or one with the partner’s genetics, were mentioned, as were reflections, conversations and the search for information on the egg donation program.

Group 2: Participants exhibiting unfavorable emotional state. This group was characterized as not demonstrating an acceptance of infertility and receiving donated eggs. The decision process was based on rational arguments and justifications but showed signs of avoiding the possible suffering and feelings that accompany the impossibility of having a biological child. Participants in this group also showed sadness or evidence of marital conflict.

Group 3: Participants without evident classification. This group consisted of participants who could not be categorized into either of the previous groups, because the time taken to discuss egg donation was not sufficient to approach all themes proposed and/or to identify the acceptance of infertility and the use of donated eggs, not meaning that the topic egg donation has not been addressed. Instead, they brought to discussion other topics also related to infertility such as treatment failures, miscarriages and loss of newborn babies.

Two years after the psychological counseling sessions, all study participants were contacted by phone to obtain information about the treatments and their outcomes - if they followed egg donation and if they have children from the treatment.

## RESULTS

Psychological counseling sessions were conducted with 43 couples, 13 women without their husbands, and 4 single women (a total of 60 sessions). The mean age of the women was 40.15 years (31 to 52 years), and the mean age of the men was 41.26 years (30 to 60 years). Concerning ethnicity, the majority of patients were white (91.66% of women and 95% of men), followed by Asian (6.66% of women and 4.65% of men) and black (1.66% of women). Concerning educational level, 83% of women and 76.74% of men had at least an undergraduate degree, and 16.66% of women and 23.25% of men had a high school education.

All participants reported that they had performed previous treatments without success. Seventeen couples reported the occurrence of miscarriages, and three couples had lost live born babies.

Twelve participants attended the psychological counseling session with a specific subject they wished to discuss, while the others did not have a specific demand.

Of the 60 psychological counseling sessions, 19 (32%) were classified as involving participants with positive emotional state (group 1), 14 (23%) involved participants with unfavorable emotional state (group 2), and 27 (45%) involved participants without evident classification (group 3).

In group 1 (favorable emotional state), 15 couples, 2 married women who came alone and 2 single women were interviewed.

One common characteristic of participants in this group was an initial refusal to use donated eggs, and some women mentioned being surprised by their doctor’s suggestion. The time between egg donation indication, acceptation and the decision to be enrolled to the program occurred after a period ranging from eight months to two years. During this period, they reported experiencing feelings of frustration because it was not possible to have a child with their own genes, which gradually diminished in intensity and caused less suffering. Questions about the procedure and feelings of guilt, incapacity, inadequacy and sadness were present. Reflections, conversations and searches for information on egg donation took place.

Nine women and one man in this group underwent psychotherapeutic follow up, and some continued therapy because they felt it helped them in the process of accepting infertility and deciding to use donated eggs. Religious faith was mentioned in ten psychological counseling sessions as an important component in the acceptance process.

In group 2 (unfavorable emotional state), 9 couples and 5 married women who came alone were considered to exhibit unfavorable emotional state for performing the treatment.

Five couples had not yet decided whether to start the treatment and mentioned different reasons, despite having subscribed to the program. Three of them showed differing opinions between the spouses, where the men did not wish to use donated eggs. Another couple showed marital conflict stemming from the difficulty of having children, which was made explicit during the psychological counseling session, and separation was considered a possible outcome of the crisis, especially by the husband. One woman in this group was distressed, sad and crying at the indication of egg donation, which had recently been made.

Participants who had decided to go through the treatment presented rational reasoning and justifications to do so, but had a tendency to minimize or deny the emotional effect of the impossibility of using their own eggs and to avoid any possible suffering. Four couples in this group decided on treatment at the time of medical indication or one month later.

During the psychological counselling sessions some women (n=4) reported that their emotional state made it difficult or impossible to perform daily activities. Three of them had experienced a recent loss, whether a miscarriage or a close relative, and were in the process of mourning the loss. Another reported that she avoided thinking about egg donation as it made her sad, and she believed that when she had a child all her problems would be solved and the egg donation would be forgotten. Three of these women reported previous treatments for depression.

Two women reported consternations regarding using donated eggs and spoke uninterruptedly during the psychological counselling session, with no possibility of establishing a dialogue in which they could reflect.

Some women in this group (n=4) lamented the impossibility of using their own eggs, making brief references to frustration, feelings of guilt, inability and impotence, accompanied by the belief that with the occurrence of pregnancy and childbirth, egg donation would be forgotten and these feelings would disappear.

Half of the women in group 2 expressed curiosity about the reaction of women who had undergone egg donation after they had given birth, questioning whether there were any reports of rejection, lack of love for the child, or postpartum depression. Some expressed concern that they would experience these reactions. Two women in this group were undergoing psychotherapy.

Most of the participants in this study (n=27) were not classified into either of the previous groups and were thus assigned to group 3. In this group, 19 couples, 6 married women who came alone and 2 single women were interviewed. Several of them used most of the psychological counselling session to discuss issues concerning intense emotions about treatment failures, miscarriages, loss of newborn babies, and questioning when to stop treatment and consider a childless life. Thus, the time taken to discuss egg donation was not sufficient to identify an acceptance process of infertility and using donated eggs.

Four participants gave short reports and seemed to be emotionally unavailable to discuss their experiences.

Five women and two men in group 3 were undergoing psychotherapeutic follow up.

### Treatment Outcome and Follow up

Three couples did not undergo treatment until two years after the psychological counselling session: one in group 1 (favorable emotional state) and two in group 2 (unfavorable emotional state). The others underwent treatment in a period ranging from one to eight months after the psychological counselling session.

In groups 1, 2 and 3, 61.11%, 75% and 51.85% of participants, respectively, had children as the result of treatment.

## DISCUSSION

The importance of childbearing and parenthood was evidenced in the reports of study participants, who over the course of a few years had performed several procedures to have a biological child. They reported histories of unsuccessful treatments, miscarriages and losses of newborn infants, in which psychological suffering was present, with repercussions on their personal, marital, social and professional lives.

For many couples who faced loss and failure, egg donation was considered a new alternative that offered increased hope of having a child (Peluffo, 2008). However, it is a second-best option with emotional repercussions that should not be minimized.

Despite the multiplicity of existing family configurations, the valorization of the nuclear family (father, mother and children) based on genetic relationships is still present in various social contexts as a normative model (Hendriks *et al*., 2017, Hammond, 2018). This family model is based on values that qualify biological family relationships as special, legitimate, more intimate and meaningful, and as natural as or better than those in which the biological connection does not exist (Mahlstedt and Greenfeld, 1989; Daniels, 2005; Hargreaves, 2006). To accept using donated eggs, which presents a new element in family configuration, beliefs and values about family need to be confronted, revised and reformulated by both members of a couple. Additionally, women are faced with the loss of a genetic connection to the child and loss of the continuation of the family bloodline, and many of them are worried they will not have an inherent bond and love their child (Greenfeld, 2015).

The process of mourning for the impossibility of having a biological child, with the associated pain and implications, is fundamental to gradually accepting a new way of becoming parents (Peluffo, 2008; Mahlstedt *et al*., 2010).

An aspect observed in participants who were classified in group 1 (favorable emotional state) was the existence of a process of questioning and personal reflection, in which positive and negative aspects of using donated eggs were considered, accompanied by feelings, fears, uncertainties and concerns. The suffering these participants experienced and their feelings of impotence and incapacity, guilt, sadness and decreased self-esteem were reported, all of which gradually diminished in intensity.

Several authors (Berger *et al*., 1986, Mahlstedt & Greenfeld, 1989; Schaffer & Diamond, 1994; Nachtigall *et al*., 1997, Van Berkel *et al*., 2007, Hersberg, 2007, Laruelle *et al*., 2011; Benward, 2015; Greenfeld, 2015) have emphasized the importance of couples getting in touch with their feelings, fears, and ambivalent thoughts about using donated gametes, as well as reflecting on the motivations that led them to choose the procedure and its implications. Mahlstedt & Greenfeld (1989) have also emphasized the importance of assessing how infertility and its treatments affect the couple as a unit and each member of the couple individually, as this will have an impact on how they address the new alternative and the resulting child.

Participants used religious and psychotherapeutic follow up, which helped them in the mourning process for the biological child and contributed to their favorable emotional state. The importance of the genetic aspect of motherhood decreased due to a reformulation of notions of parenthood. In our study, only 32% of participants were classified as having a favorable emotional state to undergo an egg donation treatment. Although it seems a small percentage of the participants, it is important to highlight that a large proportion of the patients were not able to be classify and the number of patients with favorable state could be higher.

Participants classified as group 2 (unfavorable emotional state) faced egg donation pragmatically, thinking about the advantages and disadvantages of the procedure while disregarding or minimizing the feelings related to the inability to have a biological child. Four couples chose to undergo treatment at the time egg donation was indicated or within one month of its indication. They envisioned a solution to the problem and suffering they would experience, but the problem persisted through the frustration of having a child with the genes of a woman other than the birth mother, which was different from the imagined and desired. One of these women explained during the session that she avoided thinking that the eggs would belong to another woman so as not to be sad.

The time between the medical indication and the decision to participate in the procedure is worth considering because a waiting period of several months is associated with better adjustment to the treatment chosen as it indicates a greater acceptance of infertility (Mahlstedt & Greenfeld, 1989; Sydsjö *et al.*, 2014). This acceptance reduces the likelihood that in the future, the child’s conception will be a source of suffering and family conflict (Mahlstedt & Greenfeld, 1989; Sydsjö *et al.*, 2014). According to Levinzon (2004), ambivalent feelings may surface if they have not been processed, making it difficult to cope with issues and difficulties inherent in any parental relationship.

As stated by Hargreaves (2006), the way men and women decide to undergo egg donation varies, demonstrating different ways of coping with losses, frustrations, and feelings. Although men may preserve the genetic link with the child in egg donation, it does not indicate their acceptance is free of suffering, conflicts, questions and fears, as evidenced in this study. In group 2, there were three couples with different opinions, where the men did not wish to use donated eggs. These data reinforce the importance of men’s presence in psychological counseling, since they also need to undergo a process of elaboration and acceptance in using a different means to start a family.

There is a strong recommendation that both man and woman participates in the psychological counselling sessions. Usually, the acceptance process is more difficult and slower for women, who are faced with the physical and biological limit of their bodies and the absence of a genetic link with the child. However, this does not mean that the acceptance of men is without suffering and conflict, as they have to deal with the impossibility of having a child with the genetic link of their partner and with the addition of new element in his family configuration. In this sense, the psychological counselling session with the couple can favor communication between them and the perception and understanding of their partner’s emotional state. However, some sessions are conducted only with the woman due to the impossibility or husband’s lack of interest in attending or the woman’s choice.

Some women in group 2 reported the expectation that the use of donated eggs would be forgotten during pregnancy or with the birth of the child. The desire to forget the donation seems to be related to its negation to overcome the anguish associated with being infertile and the expectation that suffering will be eliminated in an attempt to conceal the origin of the egg from themselves (Raoul-Duval *et al*., 1992, Hammond, 2018). It is worth emphasizing the difference between the desire to forget because it embodies a painful reality that has not been accepted and the desire to stop remembering, as its emotional importance has diminished during the acceptance process. For women who have accepted the infertility and the impossibility to have a biological child, it is possible that the memory of the egg donation treatment does not need to be avoided because it will not result in suffering and the anguish of impossibility.

The expectation that feelings related to egg donation will disappear during pregnancy and at child birth may be related to idealizations of motherhood and maternal love, perceived for so long as instinctive behavior (Tubert, 1996). Badinter (1985) conducted a historical study of the relationship between mother and child, demonstrating that maternal love has not always existed in the way we perceive it today. Historical, economic, cultural and subjective conditions contribute to the formation of this relationship, and therefore, it does not depend on an instinct inherent in the feminine nature. There are many ways of living with motherhood, which makes it possible to affirm that the bonds between parents and children need to be built and are based on the place that the parents designate for their children.

Curiosity about mothers’ reactions when the child is born manifested only in the women in group 2. They asked if there were reports of rejection, difficulty in bonding with the child or postpartum depression. This seemed an indication of the non-acceptance of the donated egg, projected in the future as the non-acceptance and the difficulty of bonding with a non-biological child.

Studies have been conducted with families created with donated eggs and have shown psychological wellbeing in the children, a satisfactory mother-child relationship and psychologically well-adjusted mothers (Murray *et al*., 2006; Golombok *et al*., 2011; Golombok *et al*., 2013; Blake *et al*., 2014; Bracewell-Milnes *et al*., 2016). Despite this finding, it is not a guarantee that this experience will be the same for all women.

The experience of motherhood does not always eliminate the suffering stemming from being unable to conceive one’s own child. Some mothers have unresolved grief related to infertility and the loss of biological motherhood (Hershberger *et al*., 2021). For this reason, personal processing of the impossibility of having a biological child and mourning for the loss of this child must be performed so that parents can desire and accept the child born from a donated egg and receive it as their own, regardless of the existence of a genetic link (Peluffo, 2008; Paiva, 2004; Braga & Amazonas, 2005).

In the psychological counselling session conducted, most of the couples in group 3 (without evident classification) reported in detail stories of the treatments and failures they experienced. They may have considered the sessions as an opportunity to report and share their experiences in a context in which they felt recognized without fear of being criticized and in which their suffering could be legitimized and accepted.

The suffering of infertile couples is not always recognized by family and friends because the losses suffered are not as tangible as the loss of a loved one. It must be considered that for some, infertility is experienced as a stigma in which they feel devalued and different from others, which leads them to not share this condition (Slade *et al*, 2007). Thus, these experiences tend to be lived in a solitary way, without social and emotional support, which can accentuate suffering and hinder the expression of such losses (Syme, 1997; Mahlstedt & Greenfeld, 1989).

It is worth highlighting the low adherence of patients who joined the program to psychological counselling sessions (54.54%), since they are optional. Considering the emotional complexity involved in the treatment and the future implications, it is extremely necessary a more emphatic recommendation and encouragement from the IVF healthcare professionals, emphasizing its objectives and benefits or even a broader discussion to make it mandatory for countries that still do not have in their legislation.

Benward (2015) debates the ethical dilemmas related to mandatory pretreatment counseling, limiting patients’ autonomy in deciding their participation. On the other hand, it also refers to some studies that show that patients tend to evaluate interviews positively, considering them necessary, even those who initially did not perceive their importance (Hammarberg *et al*., 2008, Hershberger *et al*., 2007).

Some countries such as the USA, United Kingdom, Australia, Belgium, New Zealand and Germany include in their fertilization programs the mandatory pretreatment counseling, having as a central aspect the discussion on disclosure donor conception for the child (Benward, 2015; Bracewell -Milnes *et al*., 2016).

This is a current hot topic, with a tendency to guide future parents on the importance of telling their child their genetic origin, encouraging them to deal with this theme in a transparent manner (Jadva *et al*., 2009; Bracewell-Milnes *et al*., 2016; Ethics Committee of the American Society for Reproductive Medicine, 2018).

Disclosure decisions are complex and based on a wide range of influences and contexts (Shehab *et al*., 2008). Some authors hypothesize that one of the aspects that can make it difficult for the parents to reveal to the child its origin is associated with non-accepted infertility and shame in needing a donated gamete. They tend to be more afraid of a child’s unfavorable reaction, and to be rejected as parents (Readings *et al*., 2011; Applegarth *et al*. 2016; Hershberger *et al*., 2021).

Obtaining the data retrospectively brought limitations to the study that must be considered. One aspect is the heterogeneity of the psychological counselling sessions with individual and couples, since different dynamics are created, which could interfere the content. Furthermore, the data obtained cannot be extrapolated to all Brazilian population, since Brazil is a continental size country, which marked regional and economic differences. This study was carried out in a private clinic, in the biggest city of Brazil and with well-educated participants. In addition, most participants were not classified according to the emotional state needed to realize the treatment, because the data that would make the classification possible were not present. These participants used psychological counseling to report infertility stories, treatments and losses. Additionally, due to the clinic protocol, sessions were not recorded, which is a procedure commonly used for qualitative studies followed by thematic analysis. This restriction made it impossible to use fragments of the participants’ reports.

Despite the limitations, this is an original study presenting data from a significant number of participants regarding the limited acceptance of donated eggs in the Brazilian population. The social, cultural and family context interferes with how the subjects deal with infertility, with the acceptance of donated gametes and with keeping or not keeping this information confidential (Nachtigall *et al*., 1997; Hershberger, 2007; Shehab et al., 2008; Laruelle *et al*., 2011).

Psychological counseling before undergoing treatment is extremely important, to access it’s emotional implications and to help couples deal with grieving and the impossibility of having a child with their own genetics, so that the child conceived without their genes is desired and accepted. This process will contribute to the mother’s wellbeing and developing satisfactory family relationships. It will also help and contribute with the decision to tell or not tell one’s child about their origin with greater tranquility and security.

## CONCLUSIONS

Couples who decide to use donor eggs establish a new element in the family constitution, which differs from what they imagined and desired when they planned to have a child.

The mourning process for the impossibility of having a biological child, with the associated pain and implications, is fundamental to gradually accepting a new way of becoming parents. Beliefs and values about family and parenthood need to be confronted, revised and reformulated.

Our study evaluated the emotional state regarding the acceptance of egg donation in couples and single women who chose to use this option in in vitro fertilization cycles. We highlighted that the decision to undergo treatment is not always associated with emotional acceptance of infertility and egg reception. In this scenario, psychological counseling is valuable to address the consideration of using donated eggs and its repercussions in family relationships, as well as to identify the need for psychotherapeutic work to address conflicts and suffering.

## References

[r1] Applegarth LD, Kaufman NL, Josephs-Sohan M, Christos PJ, Rosenwaks Z. (2016). Parental disclosure to offspring created with oocyte donation:
intentions versus reality. Hum Reprod.

[r2] Badinter E. (1985). O mito do amor materno.

[r3] Benward J. (2015). Mandatory counseling for gamete donation recipients: ethical
dilemmas. Fertil Steril.

[r4] Benyamini Y, Gozlan M, Kokia E. (2009). Women’s and men’s perceptions of infertility and their
associations with psychological adjustment: a dyadic
approach. Br J Health Psychol.

[r5] Berger DM, Eisen A, Shuber J, Doody KF. (1986). Psychological patterns in donor insemination
couples. Can J Psychiatry.

[r6] Blake L, Jadva V, Golombok S. (2014). Parent psychological adjustment, donor conception and disclosure:
a follow-up over 10 years. Hum Reprod.

[r7] Bracewell-Milnes T, Saso S, Bora S, Ismail AM, Al-Memar M, Hamed AH, Abdalla H, Thum MY. (2016). Investigating psychosocial attitudes, motivations and experiences
of oocyte donors, recipients and egg sharers: a systematic
review. Hum Reprod Update.

[r8] Braga MGR, Amazonas MCLA. (2005). Family: maternity and assisted reproduction. Psicol Estud.

[r9] Clarke V, Braun V., Teo T (2014). Encyclopedia of Critical Psychology.

[r10] Daniels K. (2005). Is blood really thicker than water? Assisted reproduction and its
impact on our thinking about family. J Psychosom Obstet Gynaecol.

[r11] Ethics Committee of the American Society for Reproductive
Medicine (2018). Informing offspring of their conception by gamete or embryo
donation: an Ethics Committee opinion. Fertil Steril.

[r12] Golombok S, Readings J, Blake L, Casey P, Mellish L, Marks A, Jadva V. (2011). Children conceived by gamete donation: psychological adjustment
and mother-child relationships at age 7. J Fam Psychol.

[r13] Golombok S, Blake L, Casey P, Roman G, Jadva V. (2013). Children born through reproductive donation: a longitudinal study
of psychological adjustment. J Child Psychol Psychiatry.

[r14] Greenfeld DA. (2015). Effects and outcomes of third-party reproduction:
parents. Fertil Steril.

[r15] Hammarberg K, Carmichael M, Tinney L, Mulder A. (2008). Gamete donors’ and recipients’ evaluation of donor conseling: a
prospective longitudinal cohort study. Aust N Z J Obstet Gynaecol.

[r16] Hammond K. (2018). The role of normative ideologies of motherhood in intended
mothers’ experiences of egg donation in Canada. Anthropol Med.

[r17] Hargreaves K. (2006). Constructing families and kinship through donor
insemination. Sociol Health Illn.

[r18] Hendriks S, Peeraer K, Bos H, Repping S, Dancet EAF. (2017). The importance of genetic parenthood for infertile men and
women. Hum Reprod.

[r19] Hershberger P, Klock SC, Barnes RB. (2007). Disclosure decisions among pregnant women who received donor
oocytes: a pphenomelogical study. Fert Steril.

[r20] Hershberger PE, Driessnack M, Kavanaugh K, Klock SC. (2021). Oocyte donation disclosure decisions: a longitudinal follow-up at
middle childhood. Hum Fertil.

[r21] Hewlett SA. (2002). Executive women and the myth of having it all. Harv Bus Rev.

[r22] Igarashi H, Takahashi T, Nagase S. (2015). Oocyte aging underlies female reproductive aging: biological
mechanisms and therapeutic strategies. Reprod Med Biol.

[r23] Indekeu A, Dierickx K, Schotsmans P, Daniels KR, Rober P, D’Hooghe T. (2013). Factors contributing to parental decision-making in disclosing
donor conception: a systematic review. Hum Reprod Update.

[r24] Jadva V, Freeman T, Kramer W, Golombok S. (2009). The experiences of adolescents and adults conceived by sperm
donation: comparisons by age of disclosure and family type. Hum Reprod.

[r25] Laruelle C, Place I, Demeestere I, Englert Y, Delbaere A. (2011). Anonymity and secrecy options of recipient couples and donors,
and ethnic origin influence in three types of oocyte
donation. Hum Reprod.

[r26] Levinzon GK. (2004). Adoção.

[r27] Luk J, Greenfeld DA, Seli E. (2010). Third party reproduction and the aging couple. Maturitas.

[r28] Mahlstedt PP. (1985). The psychological component of infertility. Fertil Steril.

[r29] Mahlstedt PP, Greenfeld DA. (1989). Assisted reproductive technology with donor gametes: the need for
patient preparation. Fertil Steril.

[r30] Mahlstedt PP, LaBounty K, Kennedy WT. (2010). The views of adult offspring of sperm donation: essential
feedback for the development of ethical guidelines within the practice of
assisted reproductive technology in the United States. Fertil Steril.

[r31] Minayo MCS. (2007). O desafio do conhecimento: pesquisa qualitativa em saúde.

[r32] Montagnini HML, Lopes HP, Straube KM, Melamed RM (2015). Temas Contemporâneos de Psicologia em Reprodução
Humana Assistida.

[r33] Montagnini HML, Malerbi F, Cedenhi AP. (2012). Egg donation and the issue of disclosure. Estud. psicol.

[r34] Murray C, MacCallum F, Golombok S. (2006). Egg donation parents and their children: follow-up at age 12
years. Fertil Steril.

[r35] Nachtigall RD, Tschann JM, Quiroga SS, Pitcher L, Becker G. (1997). Stigma, disclosure, and family functioning among parents of
children conceived through donor insemination. Fertil Steril.

[r36] Paiva LD. (2004). Adoção: significados e possibilidades.

[r37] Peluffo LU. (2008). Construcción de familias com la asistencia de gamatas
donadas. Consideraciones psicológicas, anonimato y derecho a la
identidade. Revista de Derecho de Familia.

[r38] Raoul-Duval A, Letur-Konirsch H, Frydman R. (1992). Anonymous oocyte donation: a psychological study of recipients,
donors and children. Hum Reprod.

[r39] Readings J, Blake L, Casey P, Jadva V, Golombok S. (2011). Secrecy, disclosure and everything in-between: decisions of
parents of children conceived by donor insemination, egg donation and
surrogacy. Reprod Biomed Online.

[r40] Sabatello M. (2015). Regulating Gamete Donation in the U.S.: Ethical, Legal and Social
Implications. Laws.

[r41] Schaffer JA, Diamond R., Imber-Black E (1994). Os segredos na família e na terapia familiar.

[r42] Shehab D, Duff J, Pasch LA, Mac Dougall K, Scheib JE, Nachtigall RD. (2008). How parents whose children have been conceived with donor gametes
make their disclosure decision: contexts, influences, and couple
dynamics. Fertil Steril.

[r43] Slade P, O’Neill C, Simpson AJ, Lashen H. (2007). The relationship between perceived stigma, disclosure patterns,
support and distress in new attendees at an infertility
clinic. Hum Reprod.

[r44] Sydsjö G, Lampic C, Bladh M, Skoog Svanberg A. (2014). Relationships in oocyte recipient couples - a Swedish national
prospective follow-up study. Reprod Health.

[r45] Syme GB. (1997). Facing the unacceptable: the emotional response to
infertility. Hum Reprod.

[r46] Tubert S. (1996). Mulheres sem sombra: maternidade e novas tecnologias
reprodutivas.

[r47] van Berkel D, Candido A, Pijffers WH. (2007). Becoming a mother by non-anonymous egg donation: secrecy and the
relationship between egg recipient, egg donor and egg donation
child. J Psychosom Obstet Gynaecol.

